# Mol­ecular and crystal structure of 5,9-dimethyl-5*H*-pyrano[3,2-*c*:5,6-*c*′]bis­[2,1-benzo­thia­zin]-7(9*H*)-one 6,6,8,8-tetroxide di­methyl­formamide monosolvate

**DOI:** 10.1107/S2056989019008788

**Published:** 2019-06-28

**Authors:** Andrii Rybalka, Svitlana Shishkina, Igor Ukrainets, Lyudmila Sidorenko, Galina Sim

**Affiliations:** a V. N. Karazin Kharkiv National University, 4 Svobody sq., Kharkiv 61077, Ukraine; b SSI "Institute for Single Crystals", National Academy of Sciences of Ukraine, 60, Nauky Ave., Kharkiv 61001, Ukraine; c National University of Pharmacy, 4 Valentynivska St., Kharkiv 61168, Ukraine; d Far Eastern State Medical University, 35 Murav’eva-Amurskogo St., Khabarovsk, 680000, Russian Federation

**Keywords:** benzo­thia­zine derivative, mol­ecular structure, π-stacking dimer, crystal structure

## Abstract

The title mol­ecule crystallizes as a di­methyl­formamide monosolvate, C_19_H_14_N_2_O_6_S_2_·C_3_H_7_NO. The mol­ecule was expected to adopt mirror symmetry but slightly different conformational characteristics of the condensed benzo­thia­zine ring lead to point group symmetry 1. In the crystal, mol­ecules form two types of stacking dimers with distances of 3.464 (2) Å and 3.528 (2) Å between π-systems. As a result, columns extending parallel to [100] are formed, which are connected to inter­mediate di­methyl­formamide solvent mol­ecules by C—H⋯O inter­actions.

## Abstract 2

The benzo­thia­zine skeleton is not planar, with a maximum deviation of 0.3154 (11) Å from the least-squares plane. The mol­ecule was expected to adopt mirror symmetry but slightly different conformational characteristics of the condensed benzo­thia­zine ring lead to point group symmetry 1.

## Chemical context   

Alkyl 1-*R*-4-hy­droxy-2-oxo-1,2-di­hydro­quinoline-3-carboxyl­ates are highly reactive compounds (Ukrainets *et al.*, 2007 ▸). They easily form the corresponding amides with primary and many secondary alkyl, aryl or hetaryl­amines and can be converted to 5,9-di-*R*-6,7,8-trioxodi­quinolino [3,4-*b*; 3′,4′-*e*]-4*H*-pyrans in high yields through thermolysis (Ukrainets *et al.*, 2000 ▸). The acyl­ating ability is distinctly reduced in alkyl 1-*R*-4-hy­droxy-2,2-dioxo-1*H*-2λ^6^,1-benzo­thia­zine-3-carboxyl­ates (Ukrainets *et al.*, 2014 ▸). However, a similar heterocycle, 5,9-dimethyl-5*H*-pyrano [3,2-*c*:5,6-*c*′]bis­[2,1]benzo­thia­zin-7(9*H*)-one 6,6,8,8-tetroxide (I) was synthesized based on methyl 4-hy­droxy-1-methyl-2,2-dioxo-1*H*-2λ^6^,1-benzo­thia­zine-3-carb­oxyl­ate (Ukrainets *et al.*, 2013 ▸). The mol­ecular and crystal structures of its di­methyl­formamide solvate are reported in the present communication.
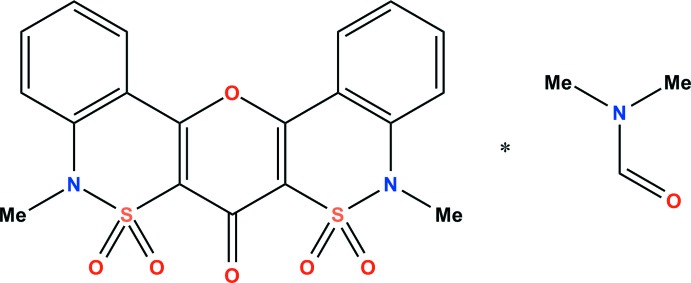


## Structural commentary   

**Figure 1:**
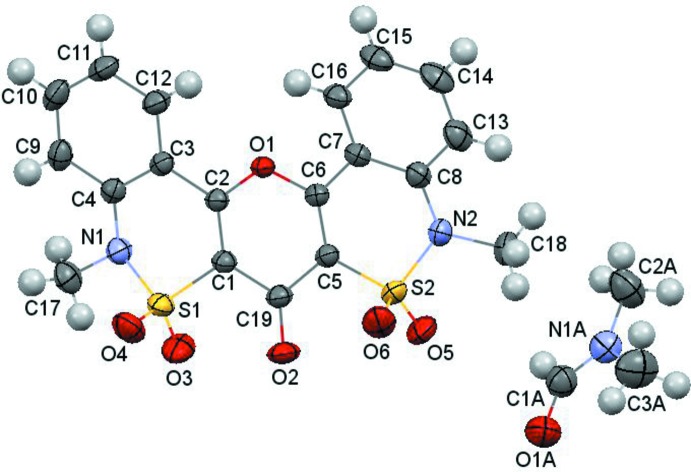
The structures of the mol­ecular entities in solvated (I). Displacement ellipsoids are drawn at the 50% probability level.

Both thia­zine rings adopt a twist-boat conformation (Fig. 1 ▸) with slightly different characteristics despite the formally mirror-symmetric mol­ecular structure of (I) in the gas phase. The puckering parameters (Zefirov *et al.*, 1990 ▸) are: *S* = 0.51, θ = 44.8°, Ψ = 28.9° for the C1–C2–C3–C4–N1–S1 ring (1) and *S* = 0.48, θ = 50.0°, Ψ = 22.8° for the C5–C6–C7–C8–N2–S2 ring (2). The S1 and C1 atoms deviate by 0.669 (2) and 0.207 (2) Å, respectively, from the mean-square plane of the remaining atoms in ring (1). The corresponding deviations in ring (2) are 0.668 (2) and 0.270 (2) Å, respectively.

The 4*H*-pyran-4-one ring (3) adopts a sofa conformation with puckering parameters *S* = 0.14, θ = 24.7°, Ψ = 22.6°. The deviation of C19 from the plane of the remaining atoms of (3) is 0.087 (2) Å. The C1=C2 and C5=C6 bonds [1.3571 (17) Å and 1.3529 (17) Å] are slightly elongated as compared to the mean value of 1.329 Å for a C*sp* ^2^=C*sp* ^2^ bond (Bürgi & Dunitz, 1994 ▸).

The mol­ecule also contains shortened contacts (the H⋯O van der Waals radii sum is 2.46 Å; Zefirov, 1997 ▸), which can be considered as attractive intra­molecular inter­actions. However, the values of the corresponding C—H⋯O angles for the pairs C17⋯O3, C18⋯O5, C3*A*⋯O1*A*, C12⋯O1, C16⋯O1 (Table 1 ▸) are too small to allow them to be characterized as intra­molecular hydrogen bonds.

A further analysis of the mol­ecular structure revealed the presence of other shortened intra­molecular contacts: H9⋯H17*C* = 2.21 Å (expected 2.34 Å), H13⋯H18*B* = 2.28 Å (expected 2.34 Å), H13⋯H18*C* = 2.31 Å (expected 2.34 Å). These shortened contacts affect the very small pyramidalization of the nitro­gen atoms; the sums of the bond angles centered at the N1 and N2 atoms are 354 and 356°, respectively.

## Supra­molecular features   

**Figure 2:**
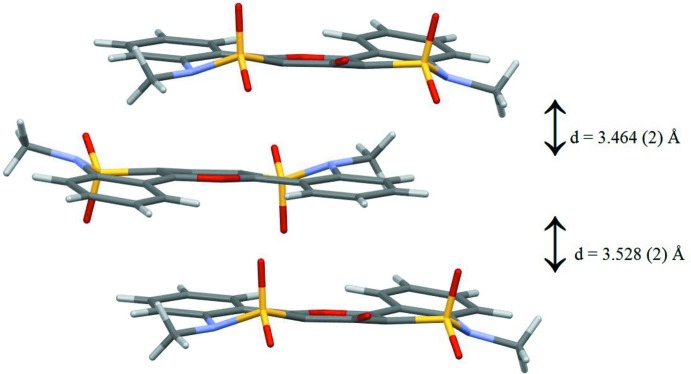
Two types of stacking dimers in the crystal structure of (I).

In the crystal, mol­ecules of (I) form columns extending parallel to [100] whereby centrosymmetric pairs of mol­ecules within a column inter­act by π–π stacking inter­actions (Fig. 2 ▸). The plane-to-plane distances between the π-systems in the centrosymmetric dimers are 3.464 (2) and 3.528 (2) Å. The mean-square plane was calculated for O1 and all carbon atoms (with the exception of C19) of the polycyclic entity.

**Figure 3:**
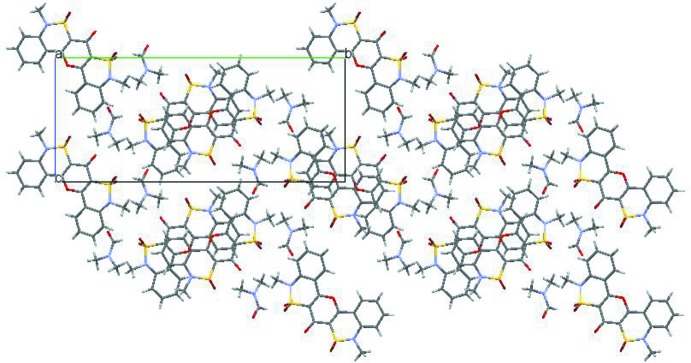
The packing of the mol­ecular entities in the crystal structure of (I) in a view along [100].

The di­methyl­formamide solvent mol­ecules are situated between the columns (Fig. 3 ▸) and are bound by weak inter­molecular hydrogen bonds including C9—H9⋯O1*A* ^i^ and C18—H18*B*⋯O1*A* ^ii^ (Table 2 ▸).

## Database survey   

A search of the Cambridge Structural Database (Version 5.38, update February 2019; Groom *et al.*, 2016 ▸) for the benzo­thia­zine skeleton revealed 34 hits. In all structures, the conformation of the benzo­thia­zine fragment is similar.

The title compound may be considered as a structural analogue of 5,9-diethyl-6,7,8-trioxodi­quinolino­[3,4-*b*;3′,4′-*e*]-4*H*-pyran (Ukrainets *et al.*, 2000 ▸) with the carbonyl groups being replaced by sulfonyl groups.

## Synthesis and crystallization   

**Figure 4:**
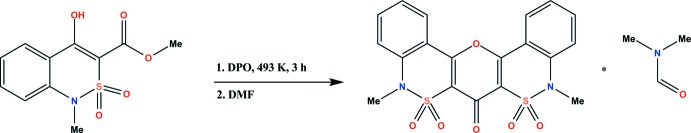
Synthesis scheme for compound (I).

A mixture of methyl 4-hy­droxy-1-methyl-2,2-dioxo-1*H*-2λ^6^,1-benzo­thia­zine-3-carboxyl­ate (2.69 g, 0.01 mol) and diphenyl oxide (10 ml) was maintained on a metal bath at 493 K for 3 h, then cooled and diluted with ethanol (Fig. 4 ▸). The precipitate was filtered off, washed with ethanol, and recrystallized from DMF. 1.86 g (37% yield) of a colourless substance were obtained, including yellowish crystals of the title solvate; m.p. 640–642 K (decomp.).

## Refinement   

Crystal data, data collection and structure refinement details are summarized in Table 2 ▸. Hydrogen atoms were located from difference-Fourier maps. They were included in calculated positions and treated as riding with C—H = 0.96 Å, *U*_iso_(H) = 1.5*U*_eq_(C) for methyl groups and with C—H = 0.93 Å, *U*_iso_(H) = 1.2*U*_eq_(C) for all other hydrogen atoms.

## Supplementary Material

Crystal structure: contains datablock(s) I. DOI: 10.1107/S2056989019008788/wm5508sup1.cif

Structure factors: contains datablock(s) I. DOI: 10.1107/S2056989019008788/wm5508Isup2.hkl

CCDC reference: 1935426

Additional supporting information:  crystallographic information; 3D view; checkCIF report

## Figures and Tables

**Table 1:** Hydrogen-bond geometry and short contacts (Å, °)

*D*—H⋯*A*	*D*—H	H⋯*A*	*D*⋯*A*	*D*—H⋯*A*
C17—H17*A*⋯O3	0.96	2.40	2.885 (2)	111
C18—H18*A*⋯O5	0.96	2.41	2.895 (2)	111
C3*A*—H3*AA*⋯O1*A*	0.96	2.41	2.784 (3)	103
C12—H12⋯O1	0.93	2.39	2.7106 (17)	100
C16—H16⋯O1	0.93	2.39	2.7109 (17)	100
C9—H9⋯O1*A* ^i^	0.93	2.41	3.324 (2)	169
C18—H18*B*⋯O1*A* ^ii^	0.96	2.46	3.376 (3)	159

Symmetry codes: (i) 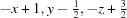; (ii) 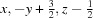.

**Table 2:** Experimental details

Crystal data	
Chemical formula	C_19_H_14_N_2_O_6_S_2_·C_3_H_7_NO
*M*_r_	503.54
Crystal system, space group	Monoclinic, *P*2_1_/*c*
Temperature (K)	293
*a*, *b*, *c* (Å)	7.2678 (2), 26.5667 (7), 11.3590 (3)
β (°)	90.498 (3)
*V* (Å^3^)	2193.13 (10)
*Z*	4
Radiation type	Mo *K*α
μ (mm^−1^)	0.30
Crystal size (mm)	0.20 × 0.20 × 0.18

Data collection	
Diffractometer	Agilent Xcalibur, Sapphire3
Absorption correction	Multi-scan (*CrysAlis RED*; Agilent, 2012 ▸)
*T*_min_, *T*_max_	0.840, 1.000
No. of measured, independent and observed [*I* > 2σ(*I*)] reflections	21958, 6370, 5409
*R*_int_	0.022
(sin θ/λ)_max_ (Å^−1^)	0.703

Refinement	
*R*[*F* ^2^ > 2σ(*F* ^2^)], *wR*(*F* ^2^), *S*	0.040, 0.111, 1.06
No. of reflections	6370
No. of parameters	311
H-atom treatment	H-atom parameters constrained
Δρ_max_, Δρ_min_ (e Å^−3^)	0.31, −0.35

Computer programs: *CrysAlis CCD* and *CrysAlis RED* (Agilent, 2012 ▸), *SHELXS* (Sheldrick, 2008 ▸), *SHELXL2016* (Sheldrick, 2015 ▸), *Mercury* (Macrae *et al.*, 2006 ▸) and *publCIF* (Westrip, 2010 ▸).

## supplementary crystallographic information

### Crystal data

**Table:**

C_19_H_14_N_2_O_6_S_2_·C_3_H_7_NO	*F*(000) = 1048
*M**_r_* = 503.54	*D*_x_ = 1.525 Mg m^−^^3^
Monoclinic, *P*2_1_/*c*	Mo *K*α radiation, λ = 0.71073 Å
*a* = 7.2678 (2) Å	Cell parameters from 8803 reflections
*b* = 26.5667 (7) Å	θ = 3.3–32.1°
*c* = 11.3590 (3) Å	µ = 0.30 mm^−^^1^
β = 90.498 (3)°	*T* = 293 K
*V* = 2193.13 (10) Å^3^	Block, colourless
*Z* = 4	0.20 × 0.20 × 0.18 mm

### Data collection

**Table:**

Agilent Xcalibur, Sapphire3 diffractometer	6370 independent reflections
Radiation source: Enhance (Mo) X-ray Source	5409 reflections with *I* > 2σ(*I*)
Detector resolution: 16.1827 pixels mm^-1^	*R*_int_ = 0.022
ω scans	θ_max_ = 30.0°, θ_min_ = 3.2°
Absorption correction: multi-scan (*CrysAlis RED*; Agilent, 2012)	*h* = −9→10
*T*_min_ = 0.840, *T*_max_ = 1.000	*k* = −34→37
21958 measured reflections	*l* = −15→15

### Refinement

**Table:**

Refinement on *F*^2^	0 restraints
Least-squares matrix: full	Hydrogen site location: inferred from neighbouring sites
*R*[*F*^2^ > 2σ(*F*^2^)] = 0.040	H-atom parameters constrained
*wR*(*F*^2^) = 0.111	*w* = 1/[σ^2^(*F*_o_^2^) + (0.056*P*)^2^ + 0.5564*P*] where *P* = (*F*_o_^2^ + 2*F*_c_^2^)/3
*S* = 1.06	(Δ/σ)_max_ = 0.001
6370 reflections	Δρ_max_ = 0.31 e Å^−^^3^
311 parameters	Δρ_min_ = −0.35 e Å^−^^3^

### Special details

**Table:**

Geometry. All esds (except the esd in the dihedral angle between two l.s. planes) are estimated using the full covariance matrix. The cell esds are taken into account individually in the estimation of esds in distances, angles and torsion angles; correlations between esds in cell parameters are only used when they are defined by crystal symmetry. An approximate (isotropic) treatment of cell esds is used for estimating esds involving l.s. planes.

### Fractional atomic coordinates and isotropic or equivalent isotropic displacement parameters (Å^2^)

**Table:**

	*x*	*y*	*z*	*U*_iso_*/*U*_eq_	
S1	0.27857 (5)	0.53088 (2)	0.78395 (3)	0.03522 (10)	
S2	0.35468 (5)	0.68735 (2)	0.47772 (3)	0.03406 (9)	
N1	0.31960 (18)	0.47039 (5)	0.76567 (10)	0.0355 (3)	
N2	0.40095 (19)	0.68763 (4)	0.33637 (11)	0.0377 (3)	
O1	0.27500 (14)	0.54076 (3)	0.43723 (8)	0.0300 (2)	
O2	0.33446 (19)	0.63741 (4)	0.71036 (9)	0.0475 (3)	
O3	0.4170 (2)	0.54940 (5)	0.86153 (10)	0.0556 (3)	
O4	0.09057 (19)	0.53887 (5)	0.81476 (11)	0.0528 (3)	
O5	0.50923 (18)	0.70840 (4)	0.53734 (11)	0.0511 (3)	
O6	0.18001 (18)	0.70953 (5)	0.50021 (12)	0.0529 (3)	
C1	0.30950 (19)	0.55350 (5)	0.64196 (11)	0.0294 (3)	
C2	0.27719 (17)	0.52260 (5)	0.54891 (11)	0.0274 (2)	
C3	0.23924 (18)	0.46944 (5)	0.55627 (12)	0.0287 (2)	
C4	0.25669 (18)	0.44458 (5)	0.66557 (12)	0.0307 (3)	
C5	0.33905 (18)	0.62303 (5)	0.50507 (11)	0.0288 (2)	
C6	0.30000 (17)	0.59081 (5)	0.41606 (11)	0.0271 (2)	
C7	0.27399 (18)	0.60439 (5)	0.29458 (11)	0.0293 (3)	
C8	0.31990 (19)	0.65325 (5)	0.25751 (12)	0.0330 (3)	
C9	0.2198 (2)	0.39295 (5)	0.67129 (15)	0.0388 (3)	
H9	0.230327	0.375995	0.742662	0.047*	
C10	0.1678 (2)	0.36734 (6)	0.57123 (16)	0.0447 (4)	
H10	0.144752	0.332967	0.575848	0.054*	
C11	0.1491 (2)	0.39142 (6)	0.46392 (16)	0.0445 (4)	
H11	0.112482	0.373420	0.397521	0.053*	
C12	0.1849 (2)	0.44209 (5)	0.45585 (13)	0.0366 (3)	
H12	0.173175	0.458353	0.383672	0.044*	
C13	0.2965 (2)	0.66588 (7)	0.13887 (14)	0.0450 (4)	
H13	0.327636	0.697916	0.112718	0.054*	
C14	0.2274 (2)	0.63092 (8)	0.06068 (14)	0.0489 (4)	
H14	0.213819	0.639567	−0.018251	0.059*	
C15	0.1778 (2)	0.58326 (7)	0.09716 (13)	0.0443 (4)	
H15	0.128749	0.560360	0.043496	0.053*	
C16	0.2012 (2)	0.56987 (6)	0.21301 (12)	0.0363 (3)	
H16	0.168641	0.537724	0.237646	0.044*	
C17	0.3401 (2)	0.44312 (7)	0.87830 (14)	0.0449 (4)	
H17C	0.418161	0.414374	0.867349	0.067*	
H17B	0.221431	0.432194	0.904561	0.067*	
H17A	0.394062	0.465053	0.936211	0.067*	
C18	0.4721 (3)	0.73589 (7)	0.29163 (18)	0.0593 (5)	
H18C	0.371718	0.755881	0.262233	0.089*	
H18B	0.557380	0.729589	0.229276	0.089*	
H18A	0.533497	0.753615	0.354267	0.089*	
C19	0.3323 (2)	0.60783 (5)	0.62857 (11)	0.0317 (3)	
N1A	0.8356 (2)	0.81500 (6)	0.40202 (13)	0.0473 (3)	
O1A	0.7557 (2)	0.81809 (6)	0.59460 (13)	0.0654 (4)	
C1A	0.8365 (3)	0.79886 (7)	0.51263 (17)	0.0503 (4)	
H1A	0.905307	0.770158	0.529054	0.060*	
C2A	0.9428 (3)	0.79092 (10)	0.3113 (2)	0.0696 (6)	
H2AC	1.005000	0.762143	0.343706	0.104*	
H2AB	1.031860	0.814287	0.281574	0.104*	
H2AA	0.862751	0.780360	0.248316	0.104*	
C3A	0.7312 (4)	0.85925 (9)	0.3696 (2)	0.0769 (7)	
H3AC	0.669591	0.853504	0.295690	0.115*	
H3AB	0.812776	0.887473	0.362385	0.115*	
H3AA	0.641725	0.866160	0.429150	0.115*	

### Atomic displacement parameters (Å^2^)

**Table:**

	*U*^11^	*U*^22^	*U*^33^	*U*^12^	*U*^13^	*U*^23^
S1	0.0477 (2)	0.03408 (17)	0.02385 (16)	−0.00072 (14)	−0.00252 (13)	−0.00174 (12)
S2	0.04352 (19)	0.02353 (15)	0.03518 (18)	0.00017 (12)	0.00303 (14)	−0.00355 (12)
N1	0.0438 (7)	0.0324 (6)	0.0301 (6)	0.0006 (5)	−0.0039 (5)	0.0038 (5)
N2	0.0502 (7)	0.0283 (6)	0.0346 (6)	−0.0031 (5)	0.0049 (5)	0.0021 (5)
O1	0.0424 (5)	0.0251 (4)	0.0226 (4)	−0.0028 (4)	0.0001 (4)	−0.0031 (3)
O2	0.0785 (9)	0.0336 (5)	0.0304 (5)	0.0003 (5)	−0.0047 (5)	−0.0108 (4)
O3	0.0812 (9)	0.0509 (7)	0.0343 (6)	−0.0151 (6)	−0.0219 (6)	0.0006 (5)
O4	0.0595 (8)	0.0560 (7)	0.0431 (6)	0.0114 (6)	0.0164 (5)	0.0001 (5)
O5	0.0641 (8)	0.0393 (6)	0.0499 (7)	−0.0184 (5)	−0.0046 (6)	−0.0075 (5)
O6	0.0597 (8)	0.0381 (6)	0.0612 (8)	0.0165 (5)	0.0154 (6)	−0.0003 (5)
C1	0.0363 (6)	0.0282 (6)	0.0237 (6)	0.0000 (5)	−0.0018 (5)	−0.0020 (5)
C2	0.0298 (6)	0.0271 (6)	0.0252 (6)	0.0003 (5)	0.0001 (4)	−0.0020 (4)
C3	0.0291 (6)	0.0258 (6)	0.0313 (6)	0.0002 (4)	0.0005 (5)	−0.0028 (5)
C4	0.0280 (6)	0.0288 (6)	0.0352 (7)	0.0016 (5)	0.0024 (5)	−0.0003 (5)
C5	0.0336 (6)	0.0251 (5)	0.0276 (6)	0.0004 (5)	0.0001 (5)	−0.0025 (5)
C6	0.0296 (6)	0.0257 (6)	0.0261 (6)	−0.0006 (4)	0.0012 (4)	−0.0018 (4)
C7	0.0304 (6)	0.0332 (6)	0.0244 (6)	0.0023 (5)	0.0008 (5)	−0.0015 (5)
C8	0.0354 (7)	0.0326 (6)	0.0311 (6)	0.0048 (5)	0.0017 (5)	0.0021 (5)
C9	0.0379 (7)	0.0301 (7)	0.0483 (8)	0.0004 (5)	0.0049 (6)	0.0069 (6)
C10	0.0441 (8)	0.0268 (6)	0.0634 (10)	−0.0044 (6)	0.0050 (7)	−0.0016 (7)
C11	0.0494 (9)	0.0331 (7)	0.0508 (9)	−0.0058 (6)	−0.0023 (7)	−0.0128 (7)
C12	0.0425 (8)	0.0310 (7)	0.0363 (7)	−0.0025 (6)	−0.0033 (6)	−0.0065 (5)
C13	0.0552 (9)	0.0445 (8)	0.0352 (8)	0.0084 (7)	0.0007 (7)	0.0103 (6)
C14	0.0537 (10)	0.0648 (11)	0.0281 (7)	0.0108 (8)	−0.0033 (6)	0.0066 (7)
C15	0.0423 (8)	0.0628 (10)	0.0278 (7)	0.0007 (7)	−0.0038 (6)	−0.0071 (7)
C16	0.0382 (7)	0.0431 (8)	0.0277 (6)	−0.0032 (6)	0.0003 (5)	−0.0058 (6)
C17	0.0520 (9)	0.0474 (9)	0.0352 (8)	0.0035 (7)	−0.0022 (7)	0.0127 (7)
C18	0.0867 (14)	0.0381 (9)	0.0533 (11)	−0.0154 (9)	0.0146 (10)	0.0071 (8)
C19	0.0398 (7)	0.0281 (6)	0.0273 (6)	0.0003 (5)	−0.0030 (5)	−0.0042 (5)
N1A	0.0451 (7)	0.0465 (8)	0.0501 (8)	0.0034 (6)	−0.0014 (6)	0.0010 (6)
O1A	0.0776 (10)	0.0597 (8)	0.0593 (8)	−0.0037 (7)	0.0145 (7)	−0.0117 (7)
C1A	0.0513 (10)	0.0458 (9)	0.0539 (10)	0.0012 (7)	0.0039 (8)	0.0010 (8)
C2A	0.0693 (13)	0.0796 (15)	0.0602 (12)	0.0119 (11)	0.0173 (10)	0.0069 (11)
C3A	0.0933 (18)	0.0628 (13)	0.0744 (15)	0.0251 (12)	−0.0108 (13)	0.0045 (11)

### Geometric parameters (Å, º)

**Table:**

S1—O3	1.4197 (12)	C10—C11	1.382 (2)
S1—O4	1.4292 (14)	C10—H10	0.9300
S1—N1	1.6479 (13)	C11—C12	1.374 (2)
S1—C1	1.7375 (13)	C11—H11	0.9300
S2—O5	1.4212 (12)	C12—H12	0.9300
S2—O6	1.4247 (12)	C13—C14	1.377 (3)
S2—N2	1.6433 (13)	C13—H13	0.9300
S2—C5	1.7405 (13)	C14—C15	1.381 (3)
N1—C4	1.4012 (18)	C14—H14	0.9300
N1—C17	1.4767 (18)	C15—C16	1.372 (2)
N2—C8	1.4052 (18)	C15—H15	0.9300
N2—C18	1.474 (2)	C16—H16	0.9300
O1—C2	1.3571 (15)	C17—H17C	0.9600
O1—C6	1.3638 (15)	C17—H17B	0.9600
O2—C19	1.2168 (16)	C17—H17A	0.9600
C1—C2	1.3571 (17)	C18—H18C	0.9600
C1—C19	1.4610 (18)	C18—H18B	0.9600
C2—C3	1.4415 (17)	C18—H18A	0.9600
C3—C12	1.4062 (18)	N1A—C1A	1.328 (2)
C3—C4	1.4110 (19)	N1A—C3A	1.445 (3)
C4—C9	1.3992 (19)	N1A—C2A	1.446 (3)
C5—C6	1.3529 (17)	O1A—C1A	1.217 (2)
C5—C19	1.4611 (18)	C1A—H1A	0.9300
C6—C7	1.4371 (17)	C2A—H2AC	0.9600
C7—C16	1.4039 (19)	C2A—H2AB	0.9600
C7—C8	1.4055 (19)	C2A—H2AA	0.9600
C8—C13	1.398 (2)	C3A—H3AC	0.9600
C9—C10	1.375 (2)	C3A—H3AB	0.9600
C9—H9	0.9300	C3A—H3AA	0.9600
			
O3—S1—O4	118.06 (9)	C12—C11—H11	120.2
O3—S1—N1	106.74 (7)	C10—C11—H11	120.2
O4—S1—N1	110.49 (7)	C11—C12—C3	120.27 (14)
O3—S1—C1	111.08 (7)	C11—C12—H12	119.9
O4—S1—C1	107.92 (7)	C3—C12—H12	119.9
N1—S1—C1	101.26 (6)	C14—C13—C8	120.03 (16)
O5—S2—O6	116.97 (8)	C14—C13—H13	120.0
O5—S2—N2	107.23 (7)	C8—C13—H13	120.0
O6—S2—N2	111.32 (8)	C13—C14—C15	121.31 (14)
O5—S2—C5	110.72 (7)	C13—C14—H14	119.3
O6—S2—C5	108.33 (7)	C15—C14—H14	119.3
N2—S2—C5	101.13 (6)	C16—C15—C14	119.65 (15)
C4—N1—C17	119.52 (12)	C16—C15—H15	120.2
C4—N1—S1	121.43 (9)	C14—C15—H15	120.2
C17—N1—S1	112.73 (10)	C15—C16—C7	120.43 (15)
C8—N2—C18	119.46 (13)	C15—C16—H16	119.8
C8—N2—S2	122.12 (10)	C7—C16—H16	119.8
C18—N2—S2	114.59 (11)	N1—C17—H17C	109.5
C2—O1—C6	120.73 (10)	N1—C17—H17B	109.5
C2—C1—C19	122.37 (12)	H17C—C17—H17B	109.5
C2—C1—S1	119.40 (10)	N1—C17—H17A	109.5
C19—C1—S1	117.00 (9)	H17C—C17—H17A	109.5
C1—C2—O1	120.90 (12)	H17B—C17—H17A	109.5
C1—C2—C3	125.40 (12)	N2—C18—H18C	109.5
O1—C2—C3	113.69 (11)	N2—C18—H18B	109.5
C12—C3—C4	119.62 (12)	H18C—C18—H18B	109.5
C12—C3—C2	120.78 (12)	N2—C18—H18A	109.5
C4—C3—C2	119.60 (12)	H18C—C18—H18A	109.5
C9—C4—N1	120.23 (13)	H18B—C18—H18A	109.5
C9—C4—C3	118.95 (13)	O2—C19—C1	124.01 (13)
N1—C4—C3	120.73 (12)	O2—C19—C5	123.66 (13)
C6—C5—C19	122.28 (12)	C1—C19—C5	112.20 (11)
C6—C5—S2	120.08 (10)	C1A—N1A—C3A	120.13 (17)
C19—C5—S2	116.45 (9)	C1A—N1A—C2A	122.24 (17)
C5—C6—O1	120.83 (11)	C3A—N1A—C2A	117.58 (18)
C5—C6—C7	125.71 (12)	O1A—C1A—N1A	126.16 (19)
O1—C6—C7	113.42 (11)	O1A—C1A—H1A	116.9
C16—C7—C8	119.64 (12)	N1A—C1A—H1A	116.9
C16—C7—C6	121.02 (12)	N1A—C2A—H2AC	109.5
C8—C7—C6	119.33 (12)	N1A—C2A—H2AB	109.5
C13—C8—N2	120.35 (14)	H2AC—C2A—H2AB	109.5
C13—C8—C7	118.91 (13)	N1A—C2A—H2AA	109.5
N2—C8—C7	120.57 (12)	H2AC—C2A—H2AA	109.5
C10—C9—C4	119.88 (15)	H2AB—C2A—H2AA	109.5
C10—C9—H9	120.1	N1A—C3A—H3AC	109.5
C4—C9—H9	120.1	N1A—C3A—H3AB	109.5
C9—C10—C11	121.62 (14)	H3AC—C3A—H3AB	109.5
C9—C10—H10	119.2	N1A—C3A—H3AA	109.5
C11—C10—H10	119.2	H3AC—C3A—H3AA	109.5
C12—C11—C10	119.67 (14)	H3AB—C3A—H3AA	109.5
			
O3—S1—N1—C4	−156.20 (12)	C19—C5—C6—O1	−8.4 (2)
O4—S1—N1—C4	74.25 (13)	S2—C5—C6—O1	−175.54 (9)
C1—S1—N1—C4	−39.91 (13)	C19—C5—C6—C7	169.19 (13)
O3—S1—N1—C17	51.92 (13)	S2—C5—C6—C7	2.0 (2)
O4—S1—N1—C17	−77.63 (12)	C2—O1—C6—C5	4.20 (19)
C1—S1—N1—C17	168.21 (11)	C2—O1—C6—C7	−173.67 (11)
O5—S2—N2—C8	154.03 (12)	C5—C6—C7—C16	−168.18 (14)
O6—S2—N2—C8	−76.87 (13)	O1—C6—C7—C16	9.57 (18)
C5—S2—N2—C8	38.02 (13)	C5—C6—C7—C8	10.9 (2)
O5—S2—N2—C18	−48.17 (15)	O1—C6—C7—C8	−171.32 (12)
O6—S2—N2—C18	80.93 (15)	C18—N2—C8—C13	−3.9 (2)
C5—S2—N2—C18	−164.18 (13)	S2—N2—C8—C13	152.83 (12)
O3—S1—C1—C2	141.18 (12)	C18—N2—C8—C7	171.24 (15)
O4—S1—C1—C2	−87.94 (13)	S2—N2—C8—C7	−32.00 (19)
N1—S1—C1—C2	28.13 (13)	C16—C7—C8—C13	−1.8 (2)
O3—S1—C1—C19	−51.17 (13)	C6—C7—C8—C13	179.13 (13)
O4—S1—C1—C19	79.71 (12)	C16—C7—C8—N2	−176.99 (13)
N1—S1—C1—C19	−164.22 (11)	C6—C7—C8—N2	3.9 (2)
C19—C1—C2—O1	3.8 (2)	N1—C4—C9—C10	−176.42 (14)
S1—C1—C2—O1	170.77 (10)	C3—C4—C9—C10	0.1 (2)
C19—C1—C2—C3	−174.90 (13)	C4—C9—C10—C11	−0.6 (2)
S1—C1—C2—C3	−7.95 (19)	C9—C10—C11—C12	0.7 (3)
C6—O1—C2—C1	−1.91 (19)	C10—C11—C12—C3	−0.4 (2)
C6—O1—C2—C3	176.96 (11)	C4—C3—C12—C11	−0.1 (2)
C1—C2—C3—C12	171.94 (13)	C2—C3—C12—C11	179.99 (14)
O1—C2—C3—C12	−6.86 (18)	N2—C8—C13—C14	176.03 (15)
C1—C2—C3—C4	−8.0 (2)	C7—C8—C13—C14	0.8 (2)
O1—C2—C3—C4	173.18 (11)	C8—C13—C14—C15	0.8 (3)
C17—N1—C4—C9	−2.2 (2)	C13—C14—C15—C16	−1.4 (3)
S1—N1—C4—C9	−152.27 (12)	C14—C15—C16—C7	0.4 (2)
C17—N1—C4—C3	−178.74 (13)	C8—C7—C16—C15	1.2 (2)
S1—N1—C4—C3	31.23 (18)	C6—C7—C16—C15	−179.73 (14)
C12—C3—C4—C9	0.2 (2)	C2—C1—C19—O2	168.80 (15)
C2—C3—C4—C9	−179.87 (13)	S1—C1—C19—O2	1.6 (2)
C12—C3—C4—N1	176.72 (13)	C2—C1—C19—C5	−7.13 (19)
C2—C3—C4—N1	−3.32 (19)	S1—C1—C19—C5	−174.38 (10)
O5—S2—C5—C6	−136.55 (12)	C6—C5—C19—O2	−166.55 (15)
O6—S2—C5—C6	93.95 (13)	S2—C5—C19—O2	1.0 (2)
N2—S2—C5—C6	−23.15 (13)	C6—C5—C19—C1	9.40 (19)
O5—S2—C5—C19	55.58 (13)	S2—C5—C19—C1	176.98 (10)
O6—S2—C5—C19	−73.91 (12)	C3A—N1A—C1A—O1A	−0.3 (3)
N2—S2—C5—C19	168.99 (11)	C2A—N1A—C1A—O1A	177.1 (2)

### Hydrogen-bond geometry (Å, º)

**Table:**

*D*—H···*A*	*D*—H	H···*A*	*D*···*A*	*D*—H···*A*
C17—H17*A*···O3	0.96	2.40	2.885 (2)	111
C18—H18*A*···O5	0.96	2.41	2.895 (2)	111
C3*A*—H3*AA*···O1*A*	0.96	2.41	2.784 (3)	103
C12—H12···O1	0.93	2.39	2.7106 (17)	100
C16—H16···O1	0.93	2.39	2.7109 (17)	100
C9—H9···O1*A*^i^	0.93	2.41	3.324 (2)	169
C18—H18*B*···O1*A*^ii^	0.96	2.46	3.376 (3)	159

Symmetry codes: (i) −*x*+1, *y*−1/2, −*z*+3/2; (ii) *x*, −*y*+3/2, *z*−1/2.
